# Commentary: Perceptual learning in autism: over-specificity and possible remedies

**DOI:** 10.3389/fnint.2016.00018

**Published:** 2016-05-25

**Authors:** Eduardo Mercado, Barbara A. Church, Amanda M. Seccia

**Affiliations:** ^1^Department of Psychology, The State University of New York, University at BuffaloBuffalo, NY, USA; ^2^Language Research Center, Georgia State UniversityAtlanta, GA, USA

**Keywords:** hyperspecificity, visual learning, autism spectrum disorder, plasticity and learning, brain plasticity and cortical reorganization

## Does repetition help or hinder learning by individuals with autism?

Approaches to treating autism often emphasize the use of intensive training to gradually improve behavior. Harris et al.'s (2015) recent report on perceptual learning by high-functioning (HF) adults with autism spectrum disorder (ASD) suggests that repetition in such interventions may actually foster inflexibility, especially in situations where individuals are trained to perform complex social behaviors. We agree that atypical learning mechanisms are an important consideration when developing behavioral interventions for ASD. However, Harris and colleagues' findings are insufficient for concluding that repetition will degrade later learning and generalization.

Historically, the effects of ASD on learning mechanisms have received much less attention than its effects on social competence, despite the fact that the behaviors most diagnostic of ASD all depend heavily on generalization of past learning (Dawson et al., 2008). Recent neuroscience studies with animal models of ASD strongly suggest that synaptic mechanisms (including synaptic plasticity) and cortical circuitry are atypical in these animals (Bourgeron, 2009, 2015); learning-related changes in neural connections are likely to also be abnormal (Leblanc and Fagiolini, 2011; Oberman et al., 2015). Visual learning tasks are known to depend on synaptic plasticity in visual cortex in typically developing (TD) animals (Cooke et al., 2015), and are associated with functional changes in V1 in TD adults (Yotsumoto et al., 2008). Recent neuroimaging studies suggest that even when adults with ASD perform similarly to TD adults after visual learning, changes in their cortical responses associated with learning may not be comparable (Schipul and Just, 2015). The fact that HF adults with ASD sometimes generalize abnormally after learning a texture discrimination task (Harris et al., 2015) further supports the hypothesis that basic learning mechanisms operate differently in individuals with ASD.

Harris et al. (2015) discovered that modifying perceptual training in ways that should reduce visual cortical adaptation improved learning and generalization by adults with ASD. They interpreted this finding as evidence that stimulus repetition during training adversely affected visual cortical processing, which in turn degraded generalization of the learned discrimination. They further speculated that similar degradation might occur in a wide range of learning contexts, and that the efficacy of behavioral interventions might be maximal only when repetition is reduced.

**Figure 1:**
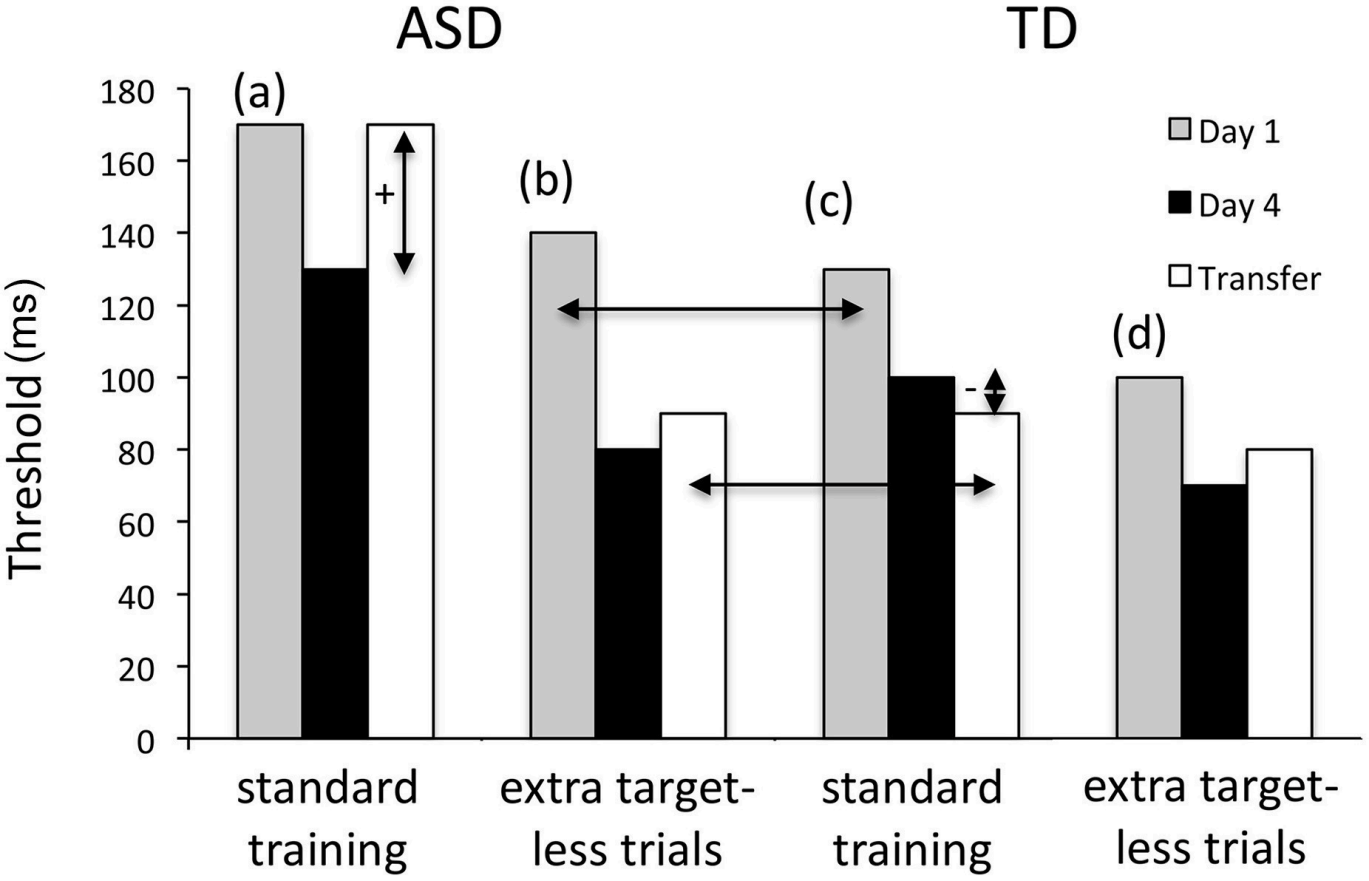
**Effects of training regimens on discrimination performance**. Harris et al. (2015) observed that HF adults with ASD (a) failed to generalize after standard training with visual textures, showing an increase in thresholds, whereas TD adults (C) generalized after standard training, showing lower thresholds. Adding extra target-less “dummy trials” led to similar generalization by both groups (b,d). They interpreted this result as evidence that reduced stimulus repetition enhanced generalization. This interpretation is confounded by between-group differences in initial performance. When initial performance was comparable across groups (b,c), learning and generalization were also comparable. An alternative account of the observed interaction is thus that generalization is degraded after training on a more difficult texture discrimination task.

While the results reported by Harris et al. (2015) clearly show that generalization of visual perceptual learning in HF adults with ASD is atypical in some training contexts and more typical in others, the mechanisms driving these training-related effects are unclear. Harris and colleagues noted that the texture discrimination task used in their past studies with TD adults (Harris et al., 2012; Harris and Sagi, 2015) had to be modified before adults with ASD were able to perform the task well; specifically, the duration of stimulus presentations was increased to make the task easier. Even using this easier version of the task, adults with ASD performed worse than TD adults after a day of training (see Figures 2A,D in Harris et al., 2015). Additionally, after 8 days of training adults with ASD were slower to respond than TD adults. Given that adults with ASD showed less overall ability to perform the texture discrimination task, an alternative interpretation of Harris et al.'s findings is that the observed between-group variations in generalization reflected differences in discrimination difficulty, rather than effects of stimulus repetition across training trials (see Figure 1). In fact, the training regimen that led to better performance, in which Harris and colleagues interspersed “dummy trials” to reduce neural adaptation to the target, contained as many target stimulus repetitions as the less effective training condition. Consequently, the number of stimulus repetitions experienced during training cannot explain any between-group differences. Adding dummy trials decreased participants' initial discrimination thresholds, but whether this effect was due to reduced neural adaptation is unclear, because the benefits of dummy trials appear to depend on how participants are pre-trained (Harris and Sagi, 2015).

Past studies of perceptual learning in TD adults (Ahissar and Hochstein, 1997; Orduña et al., 2012), and individuals with ASD (Vladusich et al., 2010; Mercado et al., 2015), have shown that variations in discrimination difficulty during training can dramatically affect generalization to novel conditions. For instance, repeatedly presenting prototypical images during a visual-category-learning task degrades generalization by TD adults, but can enhance learning and generalization by children with ASD who are having difficulty (Church et al., 2015). Similarly, how TD adults generalize after perceptual discrimination training often depends on subtle variations in regimens (Harris and Sagi, 2015). Given that individuals with ASD are highly heterogeneous in terms of their abilities, it is unlikely that any universal rule such as “avoid too much repetition during training” will maximize the benefits of learning for all individuals. Repetition that leads to frustration, boredom, or degraded neural responses obviously is non-ideal. However, repetition that consistently leads to reinforcement is one of the most powerful training approaches currently known. Individuals with ASD that experience extended training in category learning (Bott et al., 2006; Vladusich et al., 2010; Soulières et al., 2011), and sequence learning tasks (Gordon and Stark, 2007), for example, ultimately can generalize at levels comparable to TD adults. Determining when repetition during training is beneficial vs. detrimental for individuals with ASD requires a more detailed evaluation of their cognitive idiosyncrasies, such as their capacity to adjust perceptual representations through learning.

## Why understanding atypical learning in autism is critical

Harris et al. (2015) highlight an increasingly evident quality of ASD disorders—ASD does not simply disrupt social and communicative behaviors. Instead, ASD also affects a broad range of experience-dependent perceptual processes. The adults with ASD studied by Harris et al. (2015) performed worse than TD adults on a visual task that requires rapidly identifying the orientation of sets of line segments. It is unlikely that dysfunctional social abilities contributed to the difficulties that adults with ASD faced when learning to perform this task. Children and adults with ASD are processing sensory inputs (Iarocci and McDonald, 2006; Leekam et al., 2007), and motor outputs (Donnellan et al., 2012; Torres et al., 2013), in atypical ways, which undoubtedly affects how they learn about the world (Frey et al., 2013). Identifying training techniques that can aid learning by individuals with ASD requires understanding how this disorder affects both neural and perceptual plasticity.

## Author contributions

EM developed the critique and wrote the initial commentary, BC developed the critique and edited the initial commentary, AS did background research for the commentary, evaluated the critique, and edited the commentary.

### Conflict of interest statement

The authors declare that the research was conducted in the absence of any commercial or financial relationships that could be construed as a potential conflict of interest.
